# Contribution of Chronic Conditions to the Disability Burden across Smoking Categories in Middle-Aged Adults, Belgium

**DOI:** 10.1371/journal.pone.0153726

**Published:** 2016-04-22

**Authors:** Renata Tiene de Carvalho Yokota, Wilma Johanna Nusselder, Jean-Marie Robine, Jean Tafforeau, Patrick Deboosere, Herman Van Oyen

**Affiliations:** 1 Department of Public Health and Surveillance, Scientific Institute of Public Health, Rue Juliette Wytsmanstraat 14, 1050, Brussels, Belgium; 2 Department of Sociology, Interface Demography, Vrije Universiteit Brussel, Brussels, Belgium; 3 Department of Public Health, Erasmus MC, Rotterdam, Netherlands; 4 French Institute of Health and Medical Research (INSERM), Montpellier, France; 5 École Pratique des Hautes Études, Paris, France; 6 Department of Public Health, Ghent University, Ghent, Belgium; Indiana University School of Medicine, UNITED STATES

## Abstract

**Introduction:**

Smoking is considered the single most important preventable cause of morbidity and mortality worldwide, contributing to increased incidence and severity of disabling conditions. The aim of this study was to assess the contribution of chronic conditions to the disability burden across smoking categories in middle-aged adults in Belgium.

**Methods:**

Data from 10,224 individuals aged 40 to 60 years who participated in the 1997, 2001, 2004, or 2008 Health Interview Surveys in Belgium were used. Smoking status was defined as never, former (cessation ≥2 years), former (cessation <2 years), occasional light (<20 cigarettes/day), daily light, and daily heavy (≥20 cigarettes/day). To attribute disability to chronic conditions, binomial additive hazards models were fitted separately for each smoking category adjusted for gender, except for former (cessation <2 years) and occasional light smokers due to the small sample size.

**Results:**

An increasing trend in the disability prevalence was observed across smoking categories in men (never = 4.8%, former (cessation ≥2 years) = 5.8%, daily light = 7.8%, daily heavy = 10.7%) and women (never = 7.6%, former (cessation ≥2 years) = 8.0%, daily light = 10.2%, daily heavy = 12.0%). Musculoskeletal conditions showed a substantial contribution to the disability burden in men and women across all smoking categories. Other important contributors were depression and cardiovascular diseases in never smokers; depression, chronic respiratory diseases, and diabetes in former smokers (cessation ≥2 years); chronic respiratory diseases, cancer, and cardiovascular diseases in daily light smokers; cardiovascular diseases and chronic respiratory diseases in men and depression and diabetes in women daily heavy smokers.

**Conclusions:**

Beyond the well-known effect of smoking on mortality, our findings showed an increasing trend of the disability prevalence and different contributors to the disability burden across smoking categories. This information can be useful from a public health perspective to define strategies to reduce disability in Belgium.

## Introduction

The increase in life expectancy observed worldwide has raised interest not only in identifying factors associated with longevity but also with disability-free survival in an independent and healthy state [1]. Chronic diseases are the main cause of disability in late life [2–4]. In Belgium, higher disability prevalence was observed in older individuals (≥55 years) who reported at least one chronic condition (38%) compared to those individuals without any condition (13%) [5]. Older disabled individuals are at higher risk of developing complications of chronic diseases [6], resulting in poor quality of life, reduced autonomy, increased health care costs, and institutionalization [3].

Lifestyle risk factors are also part of the disablement process as they can have impact on disease, disease severity, or directly on disability [7;8]. Among the lifestyle risk factors, smoking is considered the single most important preventable cause of premature morbidity and mortality [3], yet 23% of the individuals aged 15 years or older still smoke in Belgium [9]. Smoking is associated with mortality in a greater extent than to disability possibly due to the higher risk of premature mortality in current smokers [10]. Nonetheless, the impact of smoking on disability has also been reported [11–14], mainly for being a risk factor for several chronic diseases, such as cancer, chronic respiratory diseases, and cardiovascular diseases [3] and for its exacerbating effect on existing chronic conditions [15;16]. Additionally, smoking has been identified as a predictor of mobility limitations independent of the presence of chronic conditions in middle-aged individuals [16].

Therefore, the identification of which diseases are the main contributors to the disability burden across smoking categories can assist policy-makers to define prevention strategies to reduce disability. Among the techniques previously proposed to estimate the association of chronic conditions with disability using cross-sectional data, the attribution method [17–19] has the advantage of allowing the partition of the total disability rate into individual contributions of chronic conditions, taking into account multimorbidity. As a result, investigators are able to estimate the contribution of each chronic condition to the total disability prevalence [19;20]. To date, several studies assessed the contribution of chronic diseases to the disability burden using the attribution method [17;18;20–23]. However, information is still lacking on the application of the method in a stratified analysis by smoking categories.

The aim of this study was to assess the contribution of chronic conditions to the disability burden across smoking categories in middle-aged adults in Belgium using the attribution method.

## Materials and Methods

### Study Population

In this study, data from the Health Interview Survey (HIS) conducted in Belgium in 1997, 2001, 2004, and 2008 were used. The HIS is a nationally representative household survey of the Belgian population, which includes approximately 10,000 subjects in each year. The participants were selected using a multistage, stratified (provinces and regions) and clustered (municipalities and households) sampling procedure. The response rate varied from 55% in 2008 to 61% in 2001 and 2004. Sampling weights were used to account for the differential selection probability and to reflect the composition of the Belgian population with respect to the gender and age structures. The surveys were carried out by Statistics Belgium and the Scientific Institute of Public Health. The selected households were informed that participation was voluntary via a leaflet, letter, and by the interviewer and an oral consent was obtained. Participants were informed that they were not required to answer all the questions in the survey and that they could quit anytime the interview. The researchers had no access to any identifying information from the HIS participants. The surveys were approved by the High Statistical Council in Belgium. External users can access the data with an authorisation from the Belgian Privacy Commission (https://his.wiv-isp.be/SitePages/Acces_microdata.aspx). Detailed information about the survey methodology can be found elsewhere [24;25].

This study was restricted to individuals aged 40–60 years old, as the smoking prevalence late in life is lower than in middle-aged individuals due to differential mortality effect caused by smoking and smoking cessation [10;26] and the disability prevalence at younger ages is low [10;23]. The inclusion of individuals aged 15–39 years and older than 60 years in the analysis resulted in sparseness, i.e. small sample size of current smokers aged 60 years or older and of disabled individuals aged less than 40 years (S1 Fig).

The questions about disability and chronic conditions were included in the face-to-face interview while the smoking questions were ascertained in the self-administered questionnaire. To obtain a large sample size for the analysis by smoking status, the data from four HIS were combined, resulting in a sample of 12,662 individuals aged 40 to 60 years. After excluding individuals with missing information on smoking status (N = 1,622), disability (N = 591), and chronic conditions (N = 247), 10,224 (80.7%) individuals were included in the analysis.

### Disability

Two domains were considered in our disability definition: (i) six activities of daily living (ADL)–transfer in-and-out of bed, transfer in-and-out of chair, dressing/undressing, washing hands and face, feeding, and using the toilet; and (ii) mobility limitations. The questions were not mutually exclusive. The questions were selected based on their availability in the four HIS. When asked about the ability to perform ADL tasks in the 1997, 2001, and 2004 HIS, individuals could answer: “1. No difficulty”, “2. Some difficulty”, 3. “Only with help”. In the 2008 survey, participants were asked if they usually have difficulties in performing the ADL activities by themselves. The options of answers were the same as in the previous surveys, with one extra option: “4. A lot of difficulty”. To assess mobility limitations, subjects were asked the furthest that they could walk without stopping or severe discomfort, with possible answers: “1. Only few steps”, “2. More than few steps but less than 200 meters”, or “3. 200 meters or more”. An individual was considered disabled if the answer to at least one ADL question was 2, 3, or 4 or if the answer to the mobility question was 1 or 2.

### Chronic Conditions

Six chronic conditions/groups were included in the present study: chronic respiratory diseases (asthma, chronic bronchitis, and chronic pulmonary diseases), diabetes, cancer, depression, cardiovascular diseases (ischaemic heart diseases and stroke), and musculoskeletal conditions (low back pain, osteoporosis, rheumatoid arthritis, and osteoarthritis). The reference period for each question was the year preceding the interview. These conditions were selected based on their availability in the four surveys and their contribution to the disability burden in Belgium, as shown in a previous study using the same disability definition, but without smoking stratification [23].

Some questions related to chronic conditions were modified over the four HIS. Low back pain was defined as “chronic spinal affection for longer than 3 months” in 1997; “chronic spinal condition for longer than 3 months, lumbago, sciatica, and disc prolapse” in 2001 and 2004; and “low back disorder or other chronic back problems” in 2008. In this study, low back pain was considered present in individuals who answered “yes” to the questions in any of the four HIS.

The question regarding ischaemic heart diseases was also modified over the four surveys. While “serious heart disease or heart attack” was included in the 1997, 2001, and 2004 HIS, two questions were ascertained in 2008: “myocardial infarction” and “coronary heart diseases (angina pectoris)”. In this analysis, ischaemic heart diseases were considered present in individuals who answered “yes” to the question in 1997–2004 or to at least one of the two questions in 2008.

### Smoking Exposure

Individuals were classified as never, former, occasional, and daily smokers. Former smokers were stratified according to the time since cessation as ≥2 years and <2 years. Smoking intensity was assessed for occasional and daily smokers. All occasional smokers reported being light smokers (<20 cigarettes/day) and daily smokers were further classified as light (<20 cigarettes/day) or heavy smokers (≥20 cigarettes/day).

### Statistical Analysis

The attribution method [17] was used to assess the contribution of chronic diseases to the disability burden across smoking categories. The method partitions the disability prevalence into additive contribution of chronic conditions taking into account multimorbidity and the fact that individuals can be disabled even in the absence of any disease (“background”) [17;20].

The background can represent the age effect, other disability causes that were not included in the analysis (e.g. permanent consequences of accidents), underreported and undiagnosed diseases/conditions, and the disability that is not associated with any disease/condition [18;20]. The background is present in all individuals who reported disability: in individuals who reported disability but not any of the diseases included in the analysis, disability is entirely attributed to “background”; in disabled individuals who reported one or more chronic conditions, disability is partitioned into chronic conditions and “background” [19].

In this analysis, we assumed that: (i) the distribution of disability by cause is entirely explained by the conditions that are still present at the time of the survey and the background; (ii) the cause-specific disability rates for each disease were proportionally equal in the time preceding the survey; (iii) the background rates are gender-specific; (iv) the background and disability rates are the same in the age range studied (40–60 years); (v) diseases and background act as independent competing causes; and (vi) the start of the time at risk for disability is the same for all diseases [19].

The attribution method is based on the binomial additive hazards model [17] as shown in (1).

$$\begin{array}{c} Y_{i}∼Bernoulli\left(\pi_{i}\right)\\ \pi_{i}=1-\left(\text{exp}\left(-\eta_{i}\right)\right)\\ \eta_{i}=\alpha_{g}+\sum_{d=1}^{m}\beta_{dg}(X_{di}X_{gi}) \end{array}$$ (1)

In model (1), *Y*_*i*_ is the binary response variable (disability) for each individual *i*; *π*_*i*_ is the estimated probability that individual *i* is disabled; *η*_*i*_ is the total disability rate (linear predictor) for each individual *i*; *α*_*g*_ is the background disability rate by gender *g* (0 = women, 1 = men); *β*_*dg*_ are the disease-specific disability rates (or disabling impacts) for each disease *d*(1, …, *m*) and gender *g*; *X*_*di*_ is the indicator variable for each disease *d* and individual *i*; and *X*_*gi*_ is the indicator variable for each gender *g* and individual *i*. The product (*X*_*di*_*X*_*gi*_) represents an interaction term between gender and diseases.

In this analysis, the attribution of disability to chronic conditions depends on the prevalence of each chronic condition (*X*_*di*_) and the cause-specific disability rates (*β*_*dg*_) [20].

The contribution of background and chronic conditions to the disability burden can be calculated in three steps. First, the probability of being disabled by the background (*B*_*i*_) and each chronic condition *d* (*D*_*di*_) for each individual *i* is calculated as shown in (2).

$$\begin{array}{c} B_{i}=\frac{\alpha_{g}}{\eta_{i}}\times \pi_{i}\\ D_{di}=\frac{\beta_{dg}(X_{di}X_{gi})}{\eta_{i}}\times \pi_{i} \end{array}$$ (2)

Next, the total number of disabled individuals by cause (background and chronic conditions) in each gender is obtained by summing the probability of being disabled for each cause (*B*_*i*_ and *D*_*di*_) in each gender *g*, as presented in (3).

$$\begin{array}{c} N_{bg}=\sum_{\left\{i;\;X_{gi=g}\right\}}B_{i}\\ N_{dg}=\sum_{\left\{i;\;X_{gi=g}\right\}}D_{di} \end{array}$$ (3)

In (3), *N*_*bg*_ is the total number of disabled individuals due to background in each gender *g* and *N*_*dg*_ is the total number of disabled individuals due to condition *d* in each gender *g*.

Finally, the prevalence of disability by cause in each gender *g* can be calculated as shown in (4).

$$\begin{array}{c} P_{bg}=\frac{N_{bg}}{N_{g}}\times 100\\ P_{dg}=\frac{N_{dg}}{N_{g}}\times 100 \end{array}$$ (4)

Where *P*_*bg*_ is the disability prevalence due to background in each gender *g*, *P*_*dg*_ is the disability prevalence due to condition *d* in each gender *g*, and *N*_*g*_ is the total number of individuals in each gender *g*. The total disability prevalence (*P*) is obtained by summing the *P*_*bg*_ and *P*_*dg*_ across gender.

In the results, the absolute contribution of chronic conditions and background to the disability prevalence refers to the prevalence of disability by cause defined in the formulas in (4). The relative contribution represents the percentage of the total disability prevalence (*P*_*bg*_/*P* × 100 and *P*_*dg*_/*P* × 100).

Separate models for each smoking category were fitted, but the models for former smokers (<2 years) and occasional light smokers did not converge due to the small sample size. Therefore, the results for the disability rates, contributions, and disability prevalence are restricted to never, former (≥2 years), daily light, and daily heavy smokers. Bootstrap percentile confidence intervals were estimated for the prevalence of chronic conditions, the cause-specific disability rates, and the contribution of chronic conditions to the disability prevalence by the 2.5^th^ and 97.5^th^ empirical percentiles from 1,000 bootstrap replicates sampled with replacement of equal size as the original data [27].

The statistical analyses were carried out in R, version 3.0.3 [28] using the function “BinAddHaz” in the R package “addhaz” [29] to fit the binomial additive hazards model, which is based on constrained optimization to obtain valid disability probabilities (*π*_*i*_), i.e. to ensure that the probability of being disabled (*π*_*i*_) lies between 0 and 1. More information about the attribution method can be found in previous publications [17–19].

## Results

Detailed information on the study participants are presented in Table 1. Most of the never smokers were women (61%), young (29% aged 40–44 years), and highly educated (41% with tertiary education level). Among former smokers, most of the individuals were men (59% of former smokers (≥2 years) and 56% of former smokers (< 2years)) and also highly educated (44% of former smokers (≥2 years) and 38% of former smokers (< 2years) with tertiary education level). While a higher proportion of old individuals was found in former smokers with more than 2 years since smoking cessation (31% aged 55–60 years), most of the former smokers who recently quit were young (28% aged 40–44 years). Current smokers were predominantly men, young, and with lower education level in daily smokers (tertiary level in 32% of light smokers and 29% of heavy smokers) and high education level in occasional light smokers (46% with tertiary education). Although the study population was proportionally distributed across the four survey waves, an increasing trend over time in the proportion of never smokers followed by a decrease in the proportion of former and current smokers was verified. Also, an increase in the proportion of adults who reported mobility or/and ADL limitations was observed across all across smoking categories.

**Table 1:** Characteristics of the study population. Health Interview Survey, Belgium, 1997, 2001, 2004, and 2008.

Characteristic			Smoking status											
	Total		Never		Former (≥2 years) ^a^		Former (<2 years) ^b^		Current Smokers					
									Occasional (light)		Daily			
											Light (<20 cigarettes/day)		Heavy (≥20 cigarettes/day)	
	N	%^c^	N	%^c^	N	%^c^	N	%^c^	N	%^c^	N	%^c^	N	%^c^
Gender														
Men	5056	49.5	1629	38.8	1414	59.1	178	56.2	229	56.3	769	51.8	837	58.9
Women	5168	50.5	2574	61.2	977	40.9	139	43.8	178	43.7	715	48.2	585	41.1
Age group (years)														
40–44	2782	27.2	1199	28.5	493	20.6	89	28.1	128	31.4	423	28.5	450	31.6
45–49	2530	24.7	1009	24.0	537	22.5	85	26.8	105	25.8	418	28.2	376	26.4
50–54	2351	23.0	887	21.1	632	26.4	71	22.4	88	21.6	347	23.4	326	22.9
55–60	2561	25.0	1108	26.4	729	30.5	72	22.7	86	21.1	296	19.9	270	19.0
Education level														
Tertiary	3964	38.8	1712	40.7	1050	43.9	120	37.9	187	45.9	477	32.1	418	29.4
Secondary	4661	45.6	1853	44.1	1059	44.3	141	44.5	164	40.3	741	49.9	703	49.4
Primary	1311	12.8	499	11.9	233	9.7	37	11.7	49	12.0	227	15.3	266	18.7
No diploma	82	0.8	45	1.1	7	0.3	5	1.6	0	0.0	11	0.7	14	1.0
No information	206	2.0	94	2.2	42	1.8	14	4.4	7	1.7	28	1.9	21	1.5
Survey year														
1997	2444	23.9	916	21.8	600	25.1	71	22.4	115	28.3	390	26.3	352	24.8
2001	2797	27.4	923	22.0	759	31.7	96	30.3	134	32.9	446	30.1	439	30.9
2004	2792	26.9	1229	29.2	589	24.6	98	30.9	103	25.3	334	22.5	399	28.1
2008	2231	21.8	1135	27.0	443	18.5	52	16.4	55	13.5	314	21.2	232	16.3
Mobility limitations^d^	450	4.4	170	4.0	96	4.0	17	5.4	12	2.9	78	5.3	77	5.4
Activity of daily living (ADL) limitations^e^	536	5.2	188	4.5	119	5.0	18	5.7	18	4.4	89	6.0	104	7.3
Mobility or ADL limitations	783	7.7	282	6.7	168	7.0	28	8.8	25	6.1	134	9.0	146	10.3
Mobility and ADL limitations	203	2.0	76	1.8	47	2.0	7	2.2	5	1.2	33	2.2	35	2.5

^a^Former smokers who reported smoking cessation two years or more prior to the interview.

^b^Former smokers who reported smoking cessation less than two years prior to the interview.

^c^Non-weighted proportions.

^d^Ability to walk without stopping < 200m.

^e^Difficulty in performing at least one activity of daily living task: transfer in-and-out of bed, transfer in-and-out of chair, dressing/undressing, washing hands and face, feeding, and using the toilet. The results for mobility and ADL limitations are not mutually exclusive.

**Fig 1:**
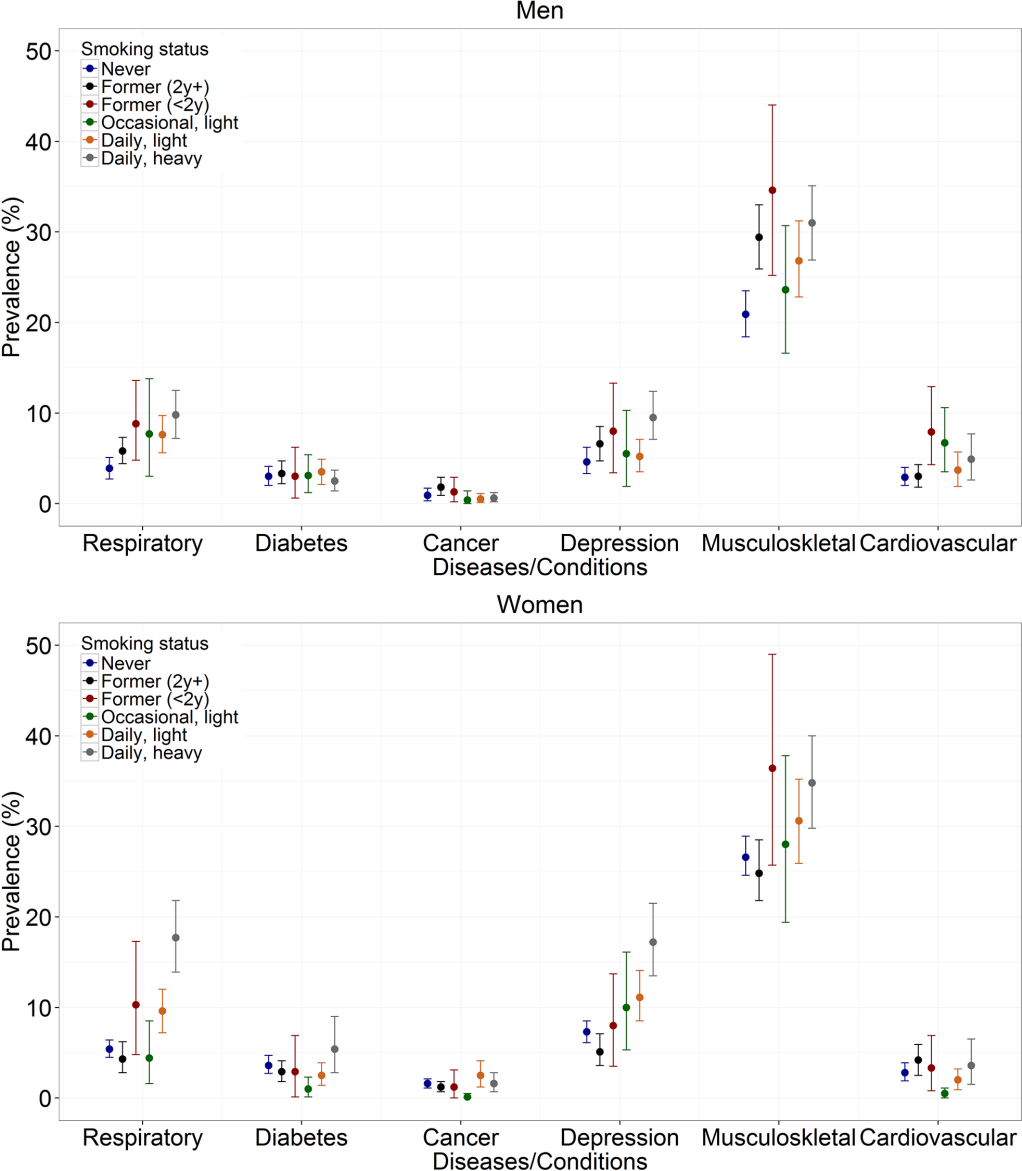
Prevalence of chronic conditions by gender and smoking categories. **Health Interview Survey, Belgium, 1997, 2001, 2004, and 2008.** Respiratory: chronic respiratory diseases (asthma, chronic bronchitis, and chronic pulmonary diseases); Musculoskeletal: musculoskeletal conditions (low back pain, osteoarthritis, rheumatoid arthritis, and osteoporosis); Cardiovascular: cardiovascular diseases (ischaemic heart diseases and stroke). Former (2y+): former smokers who reported smoking cessation two years or more prior to the interview; Former (<2y): former smokers who reported smoking cessation less than two years prior to the interview; Light: <20 cigarettes/day; Heavy: ≥20 cigarettes/day. The bars represent the bootstrap percentile confidence intervals.

Fig 1 shows the prevalence of chronic conditions in men and women across smoking categories. Musculoskeletal conditions were by far the most common conditions in men and women in all smoking categories. In men, a higher prevalence of chronic respiratory diseases and depression was observed among daily heavy smokers (9.8%; 9.5%) compared to never (3.9%; 4.6%), former (≥2 years) (5.8%; 6.6%), former (<2 years) (8.8%; 8.0%), occasional light (7.7%; 5.5%), and daily light (7.6%; 5.2%) smokers. Also, a higher prevalence of musculoskeletal conditions was observed in men heavy (31.0%) and former smokers (≥2 years: 29.4%; < 2 years: 34.6%) compared to men never smokers (20.9%). The prevalence of cardiovascular diseases was higher in men former smokers (<2 years) (7.9%) than in men never smokers (2.9%). Inversely, no difference in the prevalence of diabetes and cancer was observed across smoking categories in men. In women, the prevalence of chronic respiratory diseases and depression was much larger in heavy smokers (17.7%; 17.2%) compared to never (5.4%; 7.3%), former (≥2 years) (4.3%; 5.1%), former (<2 years) (10.3%; 8.0%), occasional light (4.4%; 10.0%), and daily light (9.6%; 11.1%) smokers. The prevalence of musculoskeletal conditions was higher in women heavy smokers (34.8%) compared to never (26.6%) and former smokers (≥ 2 years) (24.8%). The prevalence of diabetes, cancer, and cardiovascular diseases in middle aged women did not differ across smoking categories.

**Fig 2:**
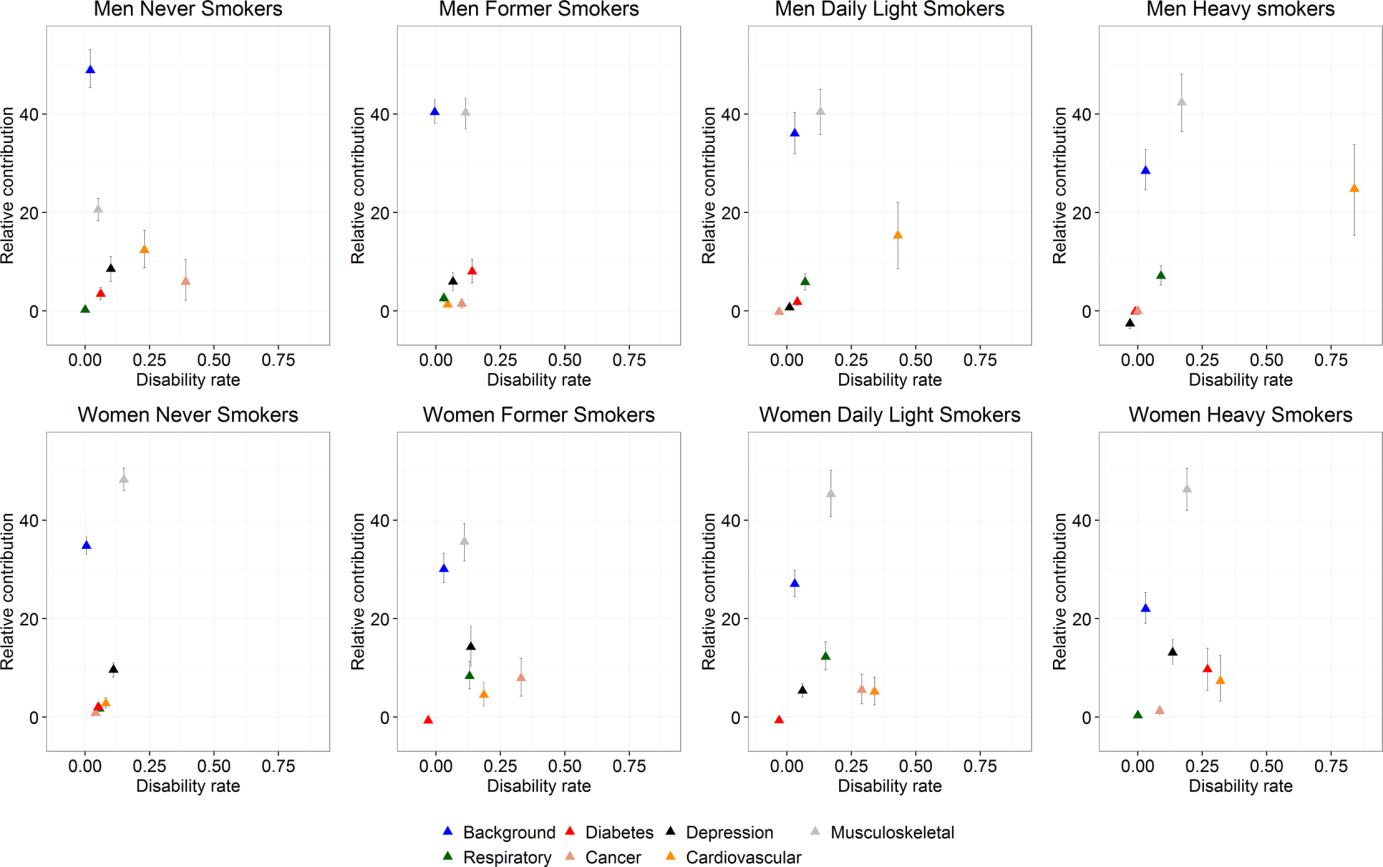
Disability rates and relative contribution of chronic conditions and background to the prevalence of disability. **Health Interview Survey, Belgium, 1997, 2001, 2004, and 2008.** The relative contribution of chronic conditions and background sum to 100%. Background: represents the diseases and conditions not included in the analysis; Respiratory: chronic respiratory diseases (asthma, chronic bronchitis, and chronic pulmonary diseases); Cardiovascular: cardiovascular diseases (ischaemic heart diseases and stroke); Musculoskeletal: musculoskeletal conditions (low back pain, osteoarthritis, rheumatoid arthritis, and osteoporosis). Former: former smokers who reported smoking cessation two years or more prior to the interview; Light: <20 cigarettes/day; Heavy: ≥20 cigarettes/day. The bars represent the bootstrap percentile confidence intervals.

The cause-specific disability rates (regression coefficients of the binomial additive hazards model) and the relative contribution of background and chronic conditions to the disability prevalence are presented in Fig 2. In men, the most disabling diseases (larger values in the “x” axis) among never smokers were cancer (0.39), cardiovascular diseases (0.23), and depression (0.10); in former smokers (≥2 years), diabetes (0.14), cancer (0.10), and depression and musculoskeletal conditions (0.09); in daily light smokers, cardiovascular diseases (0.43), musculoskeletal conditions (0.13), and chronic respiratory diseases (0.07); and in heavy smokers, cardiovascular diseases (0.84), musculoskeletal conditions (0.17) and chronic respiratory diseases (0.09). In women, the most disabling diseases in never smokers were musculoskeletal conditions (0.15), depression (0.11), and cardiovascular diseases (0.08); in former smokers (≥2 years), cancer (0.33), and depression and cardiovascular diseases (0.16); in daily light smokers, cardiovascular diseases (0.34), cancer (0.29), and musculoskeletal diseases (0.17); and in heavy smokers, cardiovascular diseases (0.32), diabetes (0.27), and musculoskeletal conditions (0.19).

**Fig 3:**
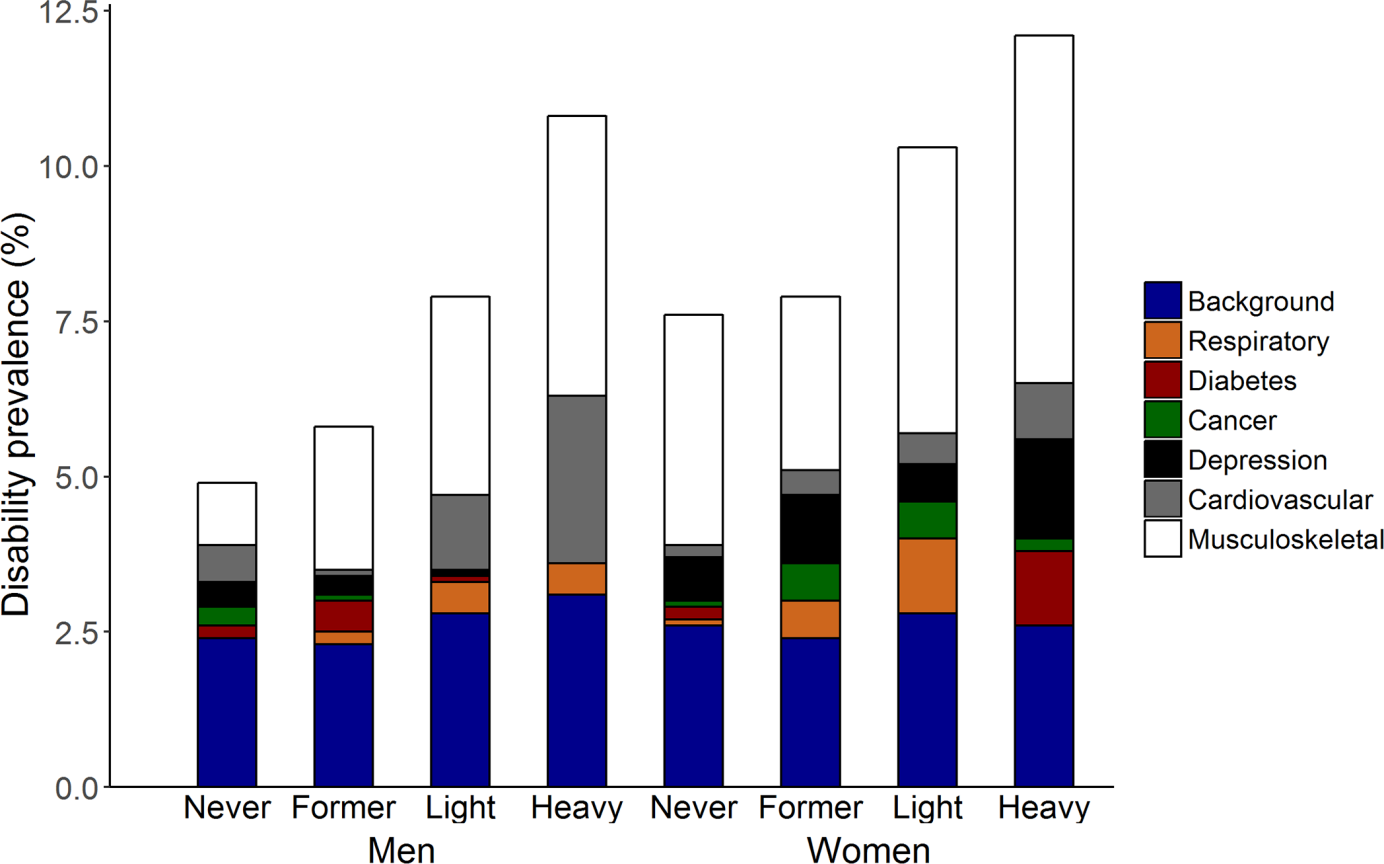
Disability prevalence and absolute contribution of chronic conditions and background to the prevalence of disability. **Health Interview Survey, Belgium, 1997, 2001, 2004, and 2008.** The absolute contribution of chronic conditions and background sum to the disability prevalence. Background: represents the diseases and conditions not included in the analysis; Respiratory: chronic respiratory diseases (asthma, chronic bronchitis, and chronic pulmonary diseases); Cardiovascular: cardiovascular diseases (ischaemic heart diseases and stroke); Musculoskeletal: musculoskeletal conditions (low back pain, osteoarthritis, rheumatoid arthritis, and osteoporosis). Former: former smokers who reported smoking cessation two years or more prior to the interview; Light: <20 cigarettes/day; Heavy: ≥20 cigarettes/day.

Musculoskeletal conditions showed the largest contribution to the disability burden in middle-aged men and women in Belgium across all smoking categories (Figs 2 and 3). Other important contributors in men included cardiovascular diseases and depression in never smokers; diabetes and depression in former smokers (≥2 years); and cardiovascular diseases and chronic respiratory diseases in daily light and heavy smokers. In women, depression and cardiovascular diseases in never smokers; depression and chronic respiratory diseases in former smokers (≥2 years); chronic respiratory diseases and cancer in daily light smokers; and depression and diabetes in heavy smokers also showed important contribution to the disability burden.

An increasing trend in the relative contribution of chronic respiratory diseases across smoking categories was observed in men (heavy = 7.1%, daily light = 5.9%, former (≥2 years) = 2.6%, never = 0.2%) and of cardiovascular diseases was observed in women (heavy = 7.3%, daily light = 5.2%, former (≥2 years) = 4.5%, never = 2.8%) (Fig 2). Despite the low background disability rates observed in all smoking categories, the background was among the top two contributors to the disability prevalence in all smoking categories (Figs 2 and 3).

An increasing trend in the prevalence of disability across smoking categories was also observed in men (never = 4.8%, 95%CI = 4.4–5.2%; former (≥2 years) = 5.8%, 95%CI = 5.5–6.2%; daily light = 7.8%, 95%CI = 7.0–8.8%; heavy = 10.7%, 95%CI = 9.4–12.3%) and women (never = 7.6%, 95%CI = 7.2–8.0%; former (≥2 years) = 8.0%, 95%CI = 7.2–8.7%; daily light = 10.2%, 95%CI = 9.3–11.2%; heavy = 12.0%, 95%CI = 10.5–13.7%) (Fig 3).

## Discussion

To our knowledge, this was the first study to focus on the contribution of chronic conditions to the disability burden across smoking categories. Our results showed that the disease prevalence, the disease-specific disability rates and the main contributors to the disability burden differ across smoking categories and gender in middle-aged adults in Belgium.

The investigation of the disability burden across smoking categories using cross-sectional data can be challenging due to the high mortality effect of smoking [30–32], the low smoking prevalence [6], and high disability prevalence at old ages. The compression of disability caused by smoking, i.e. the higher risk of premature mortality observed in smokers [10;30;31], may result in underestimation of the smoking effect on disability. In Belgium, smokers showed a reduction in life expectancy at age 30 of approximately 8 years compared to never smokers. However, the excess mortality of smokers was observed only in older ages (≥80 years), supporting the restriction of the analysis of disability stratified by smoking status to middle-aged adults [10].

Our analysis is based on the conceptual disability model proposed by Verbrugge and Jette [7], which suggests a causal link between risk factors, diseases, and disability. Although this causal relationship is plausible [2], it can only be implied and not directly assessed in cross-sectional studies. For example, the co-occurrence of smoking and depression has been widely reported in adults [33–36]. However, the causal mechanism between smoking and depression has been suggested in both directions: (i) smoking may increase the risk of depression due to a possible effect of nicotine on the neurotransmission activity of the brain; and (ii) depression may increase the risk of smoking, as it can be a form of self-medication of depressive symptoms [37]. Despite the cross-sectional nature of this study, our results are important from a public health perspective. The differences found across smoking categories can be further investigated in prospective studies.

In the attribution method, the contribution of chronic conditions to the disability prevalence is a function of the disease prevalence and the disease-specific disability rate [19;20]. Musculoskeletal conditions, depression, and chronic respiratory diseases were the most common chronic conditions across all smoking categories in men and women. A recent meta-analysis showed that smoking contributes up to 25% of the population attributable risk of rheumatoid arthritis [38]. Additionally, cotinine–a marker of tobacco exposure–was inversely associated with bone mineral content, which can contribute to the development of osteoporosis [39]. Furthermore, tobacco smoking is estimated to cause about 42% of chronic respiratory diseases [40;41].

It is interesting to note that the prevalence of cancer was low (<3%) and similar across smoking categories in men and women. This can be a result of high mortality of several types of cancer [8]. Another possible explanation is the time frame of the survey question, as the presence of self-reported cancer was restricted to the year preceding the interview. For instance, cancer diagnosis can be a reason to quit smoking, as smoking cessation is associated with increased survival after diagnosis [42]. Nonetheless, self-reported cancer cases that occurred before the year preceding the interview were not included in our analysis, as the question about ever having a chronic condition was only available for the 2008 HIS. According to the 2008 HIS, 92 individuals reported ever having cancer, of which 42 occurred in the year preceding the interview. By restricting the analysis to former smokers who quit smoking for more than 2 years in the 2008 HIS, the proportion of ever having cancer (n = 20; 4.5%) was 1.5 times larger than that of having cancer in the previous year (n = 13; 3%), suggesting that the cancer prevalence can be underestimated when defining the presence of cancer in the 12 months prior to the interview compared to ever having cancer.

Beyond the difference in the prevalence of chronic conditions across smoking categories, the most disabling conditions also differed by smoking status. Information on the cause-specific disability rate, i.e. the rate of disability among diseased individuals, can also be useful to reduce the disability burden. For example, men heavy smokers with cardiovascular diseases (β_Cardiovascular_ = 0.84; α_men_ = 0.03) have a higher probability of being disabled (π_i_ = 1–exp(–0.87) = 0.58) than men never smokers with cardiovascular diseases (β_Cardiovascular_ = 0.23; α_men_ = 0.02; π_i_ = 1–exp(–0.25) = 0.22). Likewise, women heavy smokers with cardiovascular diseases (β_Cardiovascular_ = 0.32; α_women_ = 0.03) have a higher probability of being disabled (π_i_ = 1–exp(–0.35) = 0.30) than women never smokers with cardiovascular diseases (β_Cardiovascular_ = 0.08; α_women_ = 0.03; π_i_ = 1–exp(–0.11) = 0.10). The difference in the disability probability across smoking categories suggests a possible impact of smoking on disability. In addition, the higher disability rates observed in men heavy smokers for cardiovascular diseases, musculoskeletal conditions, and chronic respiratory diseases and in women heavy smokers for cardiovascular diseases, diabetes, musculoskeletal conditions, and cancer compared to never smokers can reflect the effect of smoking on the development of chronic conditions and disability.

The most disabling conditions (high disability rate) are not necessarily the main contributors to the disability prevalence. For example, cardiovascular diseases showed the highest disability rate in women heavy smokers. However, since it was reported by few women in this subgroup (prevalence = 3.6%), the sum of the probability of being disabled by cardiovascular diseases (*D*_*Cardiovascular*,*i*_) over all women heavy smokers was low, resulting in a low relative contribution to the total disability prevalence (7.0%; 4^th^ in the rank out of 6 conditions).

One of the advantages of the attribution method is that, despite the independence assumption, the additive hazards model still takes into account multimorbidity. Analogous to the analysis of competing risks [43], the number of individuals disabled by one disease depends on the disability rates of multiple diseases. A detailed explanation about the independence assumption and its violation can be found in S1 Text.

Musculoskeletal conditions were by far the most important contributor to the disability burden across all smoking categories, mainly as a result of their high prevalence in all smoking categories. In a twin cohort study conducted in Finland [44], smokers showed a 1.7 higher risk of disability pension due to musculoskeletal conditions compared to never smokers.

The increasing trend in the relative contribution of chronic respiratory disease to the disability prevalence in men and of cardiovascular diseases in women across smoking categories also suggests an impact of smoking on the disability prevalence. Smoking can reduce lung expiratory function, resulting in a decline of ventilatory capacity. As a consequence, smokers with chronic respiratory diseases can become more readily breathless on performing ADL tasks or walking [16]. The Finish twin cohort study showed that the risk of disability retirement due to chronic obstructive pulmonary disease was increased in current (20 times) and former (3 times) smokers compared to never smokers [45]. Furthermore, smoking is among the major risk factors to cardiovascular diseases [46] and it is estimated to cause approximately 10% of cardiovascular diseases [3].

The results of this study showed a small, but increasing trend of the disability prevalence across smoking categories in men and women. In previous studies that investigated the association of smoking with physical disability [10;11;47] or functional limitations [12;48;49], small risks for current smokers compared to never smokers have also been reported. One possible explanation for this small difference is that smoking is associated in a greater extent to mortality than to disability [10;12;13;31;50]. No statistical difference was found in the prevalence of disability between women never and former smokers (≥2 years) and between women light and heavy smokers. Despite the use of a cut-off point of only 2 years to assess time since cessation, as this was the only information available in the four HIS, the similarity between women never and former smokers suggests a beneficial effect of smoking cessation longer than 2 years on disability. A previous study with middle-aged Americans showed that the risk of impaired mobility in former smokers can return to that of never smokers 15 years after quitting smoking [26]. The similar disability prevalence found in women light and heavy smokers is also relevant, since light smokers often do not consider their smoking habits as a risk behaviour [51].

This study has some limitations that should be acknowledged. Although causality is assumed by the attribution method, it cannot be confirmed with cross-sectional data. As a consequence, disability was incorrectly attributed to chronic conditions in the cases where disease developed subsequent to disability [17;20]. Selection bias might have occurred due to the relatively low response rates in the four HIS. The exclusion of individuals with missing information on chronic conditions, disability, and smoking may have underestimated the disability prevalence, owing to the overrepresentation of older and lower educated individuals among subjects with incomplete data (S1 Table).

The modification of the chronic diseases questions over the four HIS resulted in an increased prevalence of low back pain and lower prevalence of ischaemic heart diseases in 2008, as reported previously [23]. Also, under and overestimation of chronic conditions and consequently, of their contribution to the disability burden, might have occurred as a result of the use of self-reports of chronic conditions, as their validity is condition-specific [52]. Since smoking status was assessed by self-reports, misclassification may have occurred, although self-reported smoking seems to be accurate compared to biomarkers [53;54].

Furthermore, although disability has multiple dimensions [55], including instrumental activities of daily living, cognitive impairments, and sensory limitations, our disability definition was restricted to ADL and mobility limitations, which might have underestimated the disability burden in this population. Moreover, disability duration and permanence was not taken into account in our analysis.

The smoking effect on the contribution of chronic diseases to the disability burden might have been overestimated, as unhealthy behaviours such as harmful alcohol consumption, insufficient physical activity, and unhealthy diet tend to cluster within individuals. As a result, the cumulative effect of these risk factors is usually captured in cross-sectional studies [48]. Moreover, the large background contribution might indicate that important causes of disability, such as mental disorders, were not considered in this analysis [19;20;23]. These conditions were not included in this study due to their systematically unavailability in the four HIS.

The following strategies were used to cope with the limited sample size in this study:

(i) the data of the four HIS were combined, despite the difference in the smoking prevalence observed in the four surveys (S2 Fig); (ii) disability severity levels were not taken into account, although the contribution of chronic conditions to the disability burden may differ according to disability severity [56]; (iii) additional effects of disease co-occurrence, which can be measured by the interaction between chronic conditions, was not considered in the present analysis, although a synergistic effect of disease combinations has been previously reported [5]; (iv) we were not able to control the analysis by socioeconomic characteristics, which are known to be associated with disability [18;57]; and (v) the results for recent quitters (former smokers who quit for less than 2 years) and occasional light smokers were not assessed, as the models did not converge.

This study also has several strengths. First, the data used in this analysis are representative of the middle-aged Belgian population. Also, depression, which was identified as an important contributor to the disability burden in all smoking categories in women, was included in the analysis. Another added value is the assessment of the attribution of disability to chronic conditions across smoking categories, taking into account time since cessation, smoking intensity and frequency.

## Conclusions

This study has identified musculoskeletal conditions as the main contributors to the disability burden in all smoking categories in middle-aged adults in Belgium. Other diseases with high contribution in men included cardiovascular diseases and depression in never smokers; diabetes and depression in former smokers (≥2 years); and cardiovascular diseases and chronic respiratory diseases in daily light and heavy smokers. In women, other important contributors were depression and cardiovascular diseases in never smokers; depression and chronic respiratory diseases in former smokers (≥2 years); chronic respiratory diseases and cancer in daily light smokers; and depression and diabetes in heavy smokers.

Beyond the well-known health benefits of interventions aiming at smoking reduction in increasing longevity and decreasing the incidence and exacerbation of chronic conditions, they may also contribute to preserve functional status at older ages. Future research could take into account disability severity and interaction between diseases in the analysis across smoking categories and explore the role of other lifestyle risk factors, such as obesity and physical inactivity, in the estimation of cause-specific disability prevalence.

## Supporting Information

S1 Fig Prevalence of never, former, current smokers, and disability by age group and gender. **Health Interview Survey, Belgium, 1997, 2001, 2004, and 2008.** Dashed lines show the age groups included in the study (40–60 years). Current smokers include occasional and daily smokers. The bars represent the bootstrap percentile confidence intervals.
(TIF)

S2 Fig Prevalence of never, former, occasional light, daily light, and daily heavy smokers by survey year and gender in individuals aged 40–60 years. **Health Interview Survey, Belgium, 1997, 2001, 2004, and 2008.** Former (2y+): former smokers who reported smoking cessation two years or more prior to the interview; Former (<2y): former smokers who reported smoking cessation less than two years prior to the interview; Light: <20 cigarettes/day; Heavy: ≥20 cigarettes/day.
(TIF)

S1 Table Characteristics of the individuals with complete and missing information. Health Interview Survey, Belgium, 1997, 2001, 2004, and 2008. ^a^p-value obtained by the χ^2^ test.
(DOCX)

S1 Text Independence assumption and multimorbidity in the attribution method. (DOCX)
