# Supplementary figures and images for: Contribution of Chronic Conditions to the Disability Burden across Smoking Categories in Middle-Aged Adults, Belgium
Source: PLoS One. 2016 Apr 22;11(4):e0153726. doi: 10.1371/journal.pone.0153726 (PMC4841551; doi:10.1371/journal.pone.0153726)

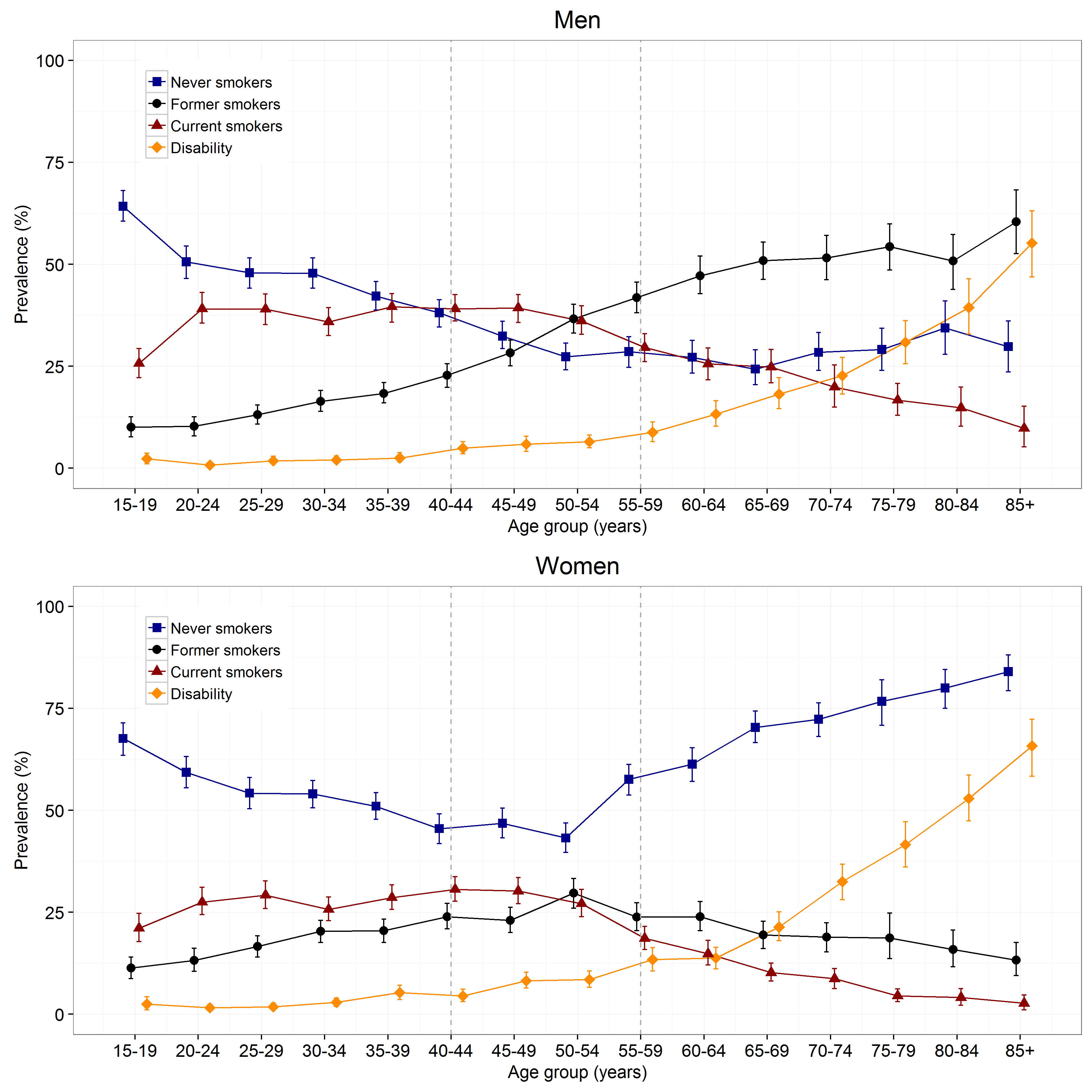

Supplement: S1 Fig — Health Interview Survey, Belgium, 1997, 2001, 2004, and 2008. Dashed lines show the age groups included in the study (40–60 years). Current smokers include occasional and daily smokers. The bars represent the bootstrap percentile confidence intervals. (TIF) [file pone.0153726.s001.tif]

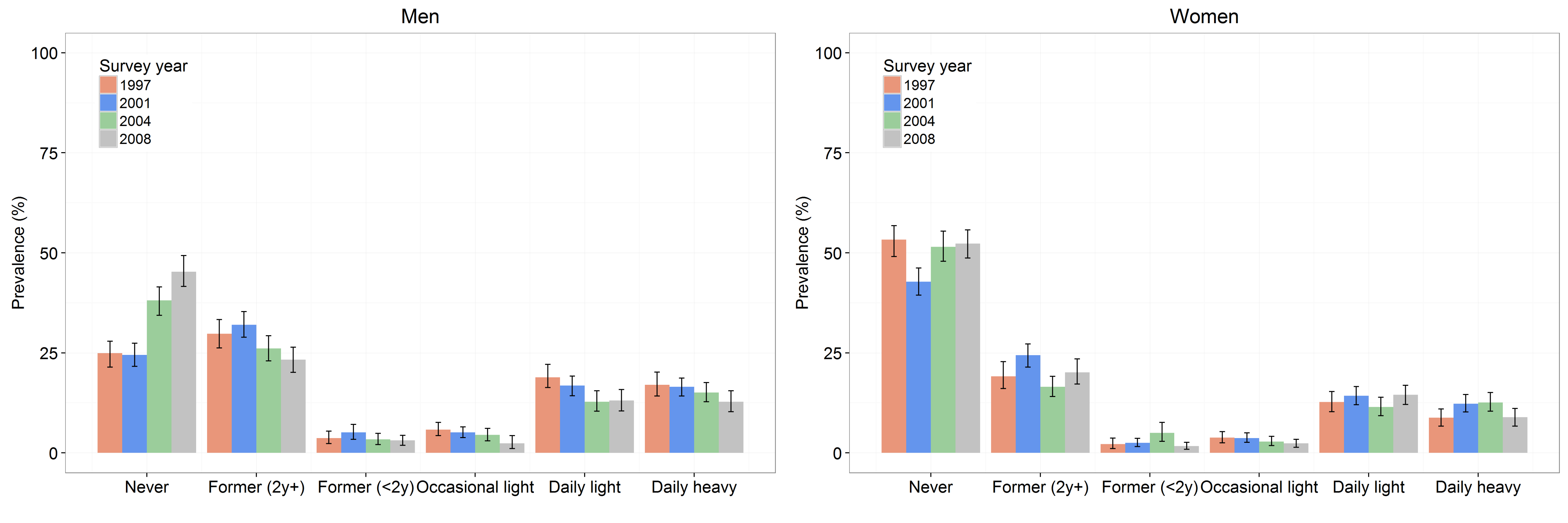

Supplement: S2 Fig — Health Interview Survey, Belgium, 1997, 2001, 2004, and 2008. Former (2y+): former smokers who reported smoking cessation two years or more prior to the interview; Former (<2y): former smokers who reported smoking cessation less than two years prior to the interview; Light: <20 cigarettes/day; Heavy: ≥20 cigarettes/day. (TIF) [file pone.0153726.s002.tif]
