# Supplementary material for: Contribution of Chronic Conditions to the Disability Burden across Smoking Categories in Middle-Aged Adults, Belgium
Source: PLoS One. 2016 Apr 22;11(4):e0153726. doi: 10.1371/journal.pone.0153726 (PMC4841551; doi:10.1371/journal.pone.0153726)
Supplement: S1 Table — Health Interview Survey, Belgium, 1997, 2001, 2004, and 2008. ap-value obtained by the χ2 test. (DOCX) [file pone.0153726.s003.docx]

**S1 Table. Characteristics of the individuals with complete and missing information. Health Interview Survey, Belgium, 1997, 2001, 2004, and 2008.**

| Characteristics | Complete | | Missing | | p-value^a^ |
| --- | --- | --- | --- | --- | --- |
|  | N | % | N | % |  |
| Gender |  |  |  |  |  |
| Male | 5056 | 49.5 | 1205 | 49.6 | 0.922 |
| Female | 5168 | 50.5 | 1225 | 50.4 |  |
| Age group (years) |  |  |  |  |  |
| 40-44 | 2782 | 27.2 | 589 | 24.2 | <0.001 |
| 45-49 | 2530 | 24.7 | 552 | 22.7 |  |
| 50-54 | 2351 | 23.0 | 583 | 24.0 |  |
| 55-60 | 2561 | 25.0 | 706 | 29.1 |  |
| Education attainment |  |  |  |  |  |
| No diploma | 82 | 0.8 | 79 | 3.3 | <0.001 |
| Primary | 1311 | 12.8 | 367 | 15.1 |  |
| Secondary | 4661 | 45.6 | 1046 | 43.0 |  |
| Tertiary | 3964 | 38.8 | 851 | 35.0 |  |
| No information | 206 | 2.0 | 87 | 3.6 |  |

^a^p-value obtained by the χ^2^ test.
