# Supplementary material for: Contribution of Chronic Conditions to the Disability Burden across Smoking Categories in Middle-Aged Adults, Belgium
Source: PLoS One. 2016 Apr 22;11(4):e0153726. doi: 10.1371/journal.pone.0153726 (PMC4841551; doi:10.1371/journal.pone.0153726)
Supplement: S1 Text — (DOCX) [file pone.0153726.s004.docx]

**S1 Text. Independence assumption and multimorbidity in the attribution method**

Even under the independence assumption, it is important to notice that the additive hazards model takes into account multimorbidity, i.e. the co-occurrence of two or more diseases in the same individual. As an illustration, consider the four groups of men heavy smokers who were classified as disabled: (i) without any reported disease; (ii) with chronic respiratory diseases (CRD); (iii) with cardiovascular diseases (CVD); and (iv) with CRD and CVD. According to the results of model, the disability rates are:

- α_men_ = 0.03
- β_CRD_ = 0.09
- β_CVD_ = 0.84

The total disability rates (η_i_) for individuals in these groups are:

- η_No_disease_ = α_men_ = 0.03
- η_CRD_ = α_men_ + β_CRD_ = 0.03 + 0.09 = 0.12
- η_CVD_ = α_men_ + β_CVD_ = 0.03 + 0.84 = 0.87
- η_CRD + CVD_ = α_men_ + β_CRD_ + β_CVD_ = 0.03 + 0.09 + 0.84 = 0.96

To interpret the rates, it is easier to convert them to probabilities (π_i_), which is done by using the exponential function:

- π_No_disease_ = 1 – exp(–η_No_disease_) = 1 – exp(–0.03) = 0.03
- π_CRD_ = 1 – exp(–η_CRD_) = 1 – exp(–0.12) = 0.11
- π_CVD_ = 1 – exp(–η_CRD_) = 1 – exp(–0.87) = 0.58
- π_CRD_ + _CVD_ = 1 – exp(–η_CRD + CVD_) = 1 – exp(–0.96) = 0.62

The probability of being disabled due to background ($B_{i}$) and to each disease ($D_{\mathrm{di}}$) can be obtained using the formulas (2) in the paper:

| $B_{i}=\frac{\alpha_{\mathrm{men}}}{\eta_{i}}\times\pi_{i}$  $D_{\mathrm{di}}=\frac{\beta_{d,men}{(X}_{\mathrm{di}}X_{men,i})}{\eta_{i}}\times\pi_{i}$ | (2) |
| --- | --- |

A summary of the results are shown in the table below:

| Group | α_men_ | β_CRD_ | β_CVD_ | η_i_ | π_i_ | B_i_ | D_CRD,i_ | D_CVD,i_ |
| --- | --- | --- | --- | --- | --- | --- | --- | --- |
| No Disease | 0.03 | 0 | 0 | 0.03 | 0.03 | 0.03 | 0 | 0 |
| CRD only | 0.03 | 0.09 | 0 | 0.12 | 0.11 | 0.03 | 0.08 | 0 |
| CVD only | 0.03 | 0 | 0.84 | 0.87 | 0.58 | 0.02 | 0 | 0.56 |
| CRD and CVD | 0.03 | 0.09 | 0.84 | 0.96 | 0.62 | 0.02 | 0.06 | 0.54 |

According to the results above, the probability of being disabled due to background ($B_{i}$), CRD ($D_{CRD,i}$), and CVD ($D_{CVD,i}$) differed across the groups, although the disability rates (α_men_, β_CRD_, β_CVD_) are the same in each group. The reason for these differences can be seen in the formulas (2), as these probabilities ($B_{i}$ and $D_{\mathrm{di}}$) depend on the total disability rate (η_i_) and total probability of being disabled (π_i_). In this example, we can clearly see that the probability of being disabled due to one disease depends on the presence of other diseases, which is analogous to the mortality analysis of competing risks. In this example we still assume independence, but multimorbidity is taken into account.

The violation of the independence assumption in the example above could be represented by a two-way interaction between CRD and CVD. Suppose that the interaction term between these two diseases was estimated by the model and it was significant (β_CRD*CVD_ = 1.2). We could re-write the last row of the table above by adding extra columns for the interaction term:

- η_CRD + CVD_ = α_men_ + β_CRD_ + β_CVD_ + β_CRD*CVD_ = 0.03 + 0.09 + 0.84 + 1.2 = 2.2
- π_CRD_ + _CVD_ = 1 – exp(–η_CRD + CVD_) = 1 – exp(–2.2) = 0.89

| Group | α_men_ | β_CRD_ | β_CVD_ | **β_CRD*CVD_** | η_i_ | π_i_ | B_i_ | D_CRD,i_ | D_CVD,i_ | **D_CRD*CVD,i_** |
| --- | --- | --- | --- | --- | --- | --- | --- | --- | --- | --- |
| CRD and CVD | 0.03 | 0.09 | 0.84 | **1.2** | 2.2 | 0.89 | 0.01 | 0.04 | 0.34 | **0.49** |

The results indicate that the disability rate due to the co-occurrence of CRD and CVD (β_CRD*CVD_  = 1.2) is larger than the sum of the individual (main) effects of these diseases (β_CRD_ + β_CVD_=0.93); i.e., a synergistic effect of the co-occurrence of these diseases is observed.
